# A collaboration framework to translate academic research to drug development

**DOI:** 10.1002/alz70859_096025

**Published:** 2025-12-25

**Authors:** Fiona E Jeganathan, Alzheimer's Research UK, Drug Discovery Institute, University College London

**Affiliations:** ^1^ ARUK Drug Discovery Unit University College London, London, London United Kingdom

## Abstract

A collaboration framework to translate academic research to drug development.

At the Alzheimer’s Research UK Drug Discovery Institute at University College London (ARUK UDDI), we bridge the gap between academic discoveries and the pharmaceutical industry by transforming cutting‐edge discoveries in the field of dementia and neurodegeneration into potential therapeutic candidates. In this session, we will discuss how by partnering with academic innovators and combining industry capabilities and talent across biology, pharmacology and screening, we can accelerate novel discoveries towards potential treatments for Alzheimer’s disease and related dementias.

We take biological targets for human disease that have data confirming their role in human disease and then validate these targets through rigorous preclinical studies. We then design and develop high throughput screening assays to identify small molecules that modulate their activity. Using advanced screening platforms, medicinal chemistry, and in vitro and in vivo models, we optimise lead compounds for safety, efficacy, and drug‐like properties and position projects for development in industry. We prioritise biomarker discovery in the early stages of our projects to ensure our projects to support preclinical and clinical trial progression.

Our operating model emphasises collaboration and open innovation. We partner using a broad variety of contractual frameworks with academics across and beyond UCL, pharmaceutical and biotechnology companies and investors to build the data sets to drive our portfolio projects towards patients with the goal of delivering a toolbox of treatments for patients throughout their dementia journey.

